# Co-prescription of metformin and antipsychotics in severe mental illness: a UK primary care cohort study

**DOI:** 10.1136/bmjment-2024-301505

**Published:** 2025-04-02

**Authors:** Luiza Farache Trajano, Joseph F Hayes, Naomi Launders, Neil M Davies, David P J Osborn, Alvin Richards-Belle

**Affiliations:** 1 Division of Psychiatry, University College London, London, UK; 2 North London NHS Foundation Trust, London, UK; 3 Department of Statistical Science, University College London, London, UK

**Keywords:** adult psychiatry, schizophrenia & psychotic disorders, psychiatry, data interpretation, statistical

## Abstract

**ABSTRACT:**

**Background:**

Metformin is a pharmacological candidate to mitigate second-generation antipsychotic (SGA)-induced weight gain in patients diagnosed with severe mental illnesses (SMI).

**Objective:**

To determine the incidence, prevalence and demographic patterns of metformin co-prescription among patients diagnosed with SMI initiating SGAs. To estimate the impact of metformin co-prescription on weight over 2 years post-SGA initiation.

**Methods:**

A cohort study of patients diagnosed with SMI initiating aripiprazole, olanzapine, quetiapine or risperidone in 2005–2019 using primary care data from Clinical Practice Research Datalink. We estimated cumulative incidence and period prevalences of co-prescription and explored prescribing differences by demographic and clinical factors. We compared weight change among patients prescribed an SGA-only versus an SGA plus metformin, accounting for confounders using linear regression.

**Findings:**

Among 26 537 patients initiating SGAs, 4652 were ever prescribed metformin and 21 885 were not. The two-year incidence of first metformin prescription was 3.3%. The SGA plus metformin group were more ethnically diverse, had greater social deprivation, more comorbidities and higher baseline weight (mean 90.4 vs 76.8 kg). By 2 years post-SGA initiation, mean weight in the SGA-only group had changed by +4.16% (95% CI −1.26 to +9.58) compared with −0.65% (95% CI −4.26 to +2.96) in the SGA plus metformin group. After confounder adjustment, the 2-year mean difference in weight with metformin co-prescription was −1.48 kg (95% CI −4.03 to 1.07) among females and −1.84 kg (95% CI −4.67 to 0.98) among males.

**Conclusion:**

Metformin is infrequently co-prescribed, despite apparent efficacy and guidelines.

**Clinical implications:**

Primary and secondary care collaboration should be strengthened and barriers to co-prescribing addressed.

**WHAT IS ALREADY KNOWN ON THIS TOPIC**

**WHAT THIS STUDY ADDS**

- This study describes real-world co-prescribing patterns of metformin and SGAs in UK primary care.
- We demonstrate that metformin is infrequently co-prescribed yet it may contribute to mitigating weight gain; however, firm conclusions about its effect on weight change could not be drawn, possibly due to the small number of co-prescriptions and the heterogeneity of the data.

**HOW THIS STUDY MIGHT AFFECT RESEARCH, PRACTICE OR POLICY**

- This study underscores the need to explore barriers to metformin co-prescription for patients diagnosed with severe mental illnesses.
- It also emphasises the importance of developing clear prescribing guidelines to support primary and secondary care clinicians in managing antipsychotic-induced weight gain.
- Further well-powered studies are needed to reliably estimate the real-world effect of metformin co-prescription on weight change.

## Background

 Second-generation antipsychotic (SGA) medications are effective for many patients in the treatment of severe mental illness (SMI), including schizophrenia and bipolar disorder.^1^,^3^ In the UK, four SGAs—olanzapine, quetiapine, risperidone and aripiprazole—account for 79% of all antipsychotic prescriptions for patients diagnosed with SMI.^4^ These SGAs can have a significant adverse effect profile, including weight gain, particularly with olanzapine, quetiapine and risperidone.^5 6^ Although aripiprazole is generally associated with lower metabolic risk, some individuals experience significant weight gain.^7^ During the first 3 years following SGA initiation, 80% of patients with first-episode psychosis experience clinically significant weight gain (≥7% of their baseline body weight).^8^ Antipsychotic-induced weight gain (AIWG) leads to an increased risk of cardiovascular and metabolic conditions, including myocardial infarction, cerebrovascular accidents, dyslipidaemia (abnormal lipid levels) and diabetes.^5 9 10^ Moreover, AIWG can lead to psychological distress, non-adherence and early antipsychotic discontinuation.^8 11^

Non-pharmacological approaches, such as cognitive and behavioural interventions, physical activity and dietary modifications, are effective at managing AIWG for some, but not all, patients.^12 13^ Randomised clinical trials (RCTs) investigating the efficacy of these non-pharmacological interventions have suffered high attrition, indicative of adherence challenges.^14^ Metformin hydrochloride (metformin), a glucose-lowering medication, licensed for managing type 1 and type 2 diabetes mellitus and polycystic ovary syndrome (PCOS),^15^ has demonstrated significant efficacy in the management of AIWG,^16^,^18^ outperforming other pharmacological candidates in RCTs and meta-analyses,^19 20^ although the role of glucagon-like peptide-1 (GLP-1) receptor agonists is yet to be determined. In 2016, the British Association for Psychopharmacology (BAP) recommended adjunctive metformin to manage AIWG and reduce diabetes risk.^21^ A 2023 UK National Institute of Health and Care Research (NIHR) call for research into the clinical and cost-effectiveness of metformin in preventing AIWG in first-episode psychosis highlighted a national need for further research in this area.^22^ In 2024, the National Institute for Health and Care Excellence (NICE) reviewed 18 studies and concluded that metformin consistently reduces AIWG by approximately 3 kg (range −2.5 to −4) when compared with placebo.^23^ This NICE surveillance review recommended that the NICE guidelines on psychosis and schizophrenia in adults^24^ are updated to ‘explore the role of metformin for managing AIWG’, including off-label use.^23^ Consensus guidelines on the co-commencement of metformin with antipsychotics to prevent AIWG were also published.^25^ However, despite an accruing evidence base and guideline recommendations, the extent to which metformin is currently being used to manage AIWG in the UK is unclear. Understanding current practice might help to inform the implementation of guideline recommendations and highlight unmet need.

### Aim and objectives

In this study, we aimed to determine patterns of co-prescribing of SGAs and metformin for people diagnosed with SMI in UK primary care, initiating an SGA between 1 January 2005 and 31 December 2017. Our specific objectives were as follows:

1. To describe, among patients newly prescribed aripiprazole, olanzapine, quetiapine or risperidone, the cumulative incidence and prevalence of metformin co-prescription.
2. To describe the co-prescription of metformin and SGAs according to demographic and clinical factors and explore if these factors differ by potential metformin indication.
3. To describe changes in weight up to 2 years post-SGA initiation among patients co-prescribed metformin plus an SGA versus patients prescribed an SGA only and to estimate the effect of metformin co-prescription on weight change.

## Methods

### Study design

We conducted an observational longitudinal cohort study to investigate SGA and metformin co-prescribing in UK primary care from 1 January 2005 to 31 December 2019.

### Data source

We used data from the Clinical Practice Research Datalink (CPRD), which includes two anonymised databases: GOLD^26^ and Aurum.^27^ These databases include current and historical primary care records for >62 million patients. We used the April 2023 and May 2022 database builds of GOLD and Aurum, respectively. For patients registered at primary care practices in England, CPRD data are linked to hospitalisation records (Hospital Episode Statistics) and to area-level deprivation data (the 2019 English Index of Multiple Deprivation (IMD)).

### Population

The study cohort included patients newly prescribed aripiprazole, olanzapine, quetiapine or risperidone in primary care—the four most frequently prescribed antipsychotics in the UK^4^—and has been previously characterised.^28^ Inclusion criteria, applied as at the date of first prescription for the SGA, were: age 18–99 years; SMI diagnosis recorded in primary care (ie, schizophrenia, bipolar disorder or other non-organic psychoses (eg, schizoaffective disorders, delusional disorder, psychotic episodes, non-organic psychosis not otherwise specified)); primary care practice registration for at least 6 months and at least one lipid or glycated haemoglobin (HbA1c) measurement recorded in the last 2 years. Exclusion criteria were: prescription of more than one antipsychotic (or a long-acting injectable antipsychotic in the prior 90 days) and dementia diagnosis.

Patients meeting eligibility criteria entered the study on their first prescription (index) date between 1 January 2005 and 31 December 2017 and completed the study at the earliest of: completion of 2-year follow-up (final follow-up, 31 December 2019), end of primary care registration, death or the last data collection date from the primary care practice. Further cohort details and other outcomes are reported in the study by Richards-Belle *et al*.^28^

### Data management

To identify antipsychotic^4^ and metformin (online supplemental table 1) prescriptions, we developed search strategies (which considered generic and brand names) to identify relevant product codes in CPRD product code dictionaries. We used the resulting code lists to extract data on these medications from patient prescription records.

To characterise the cohort, we extracted data on the following demographic and baseline characteristics: sex, ethnicity, SMI diagnosis, age (at first SMI diagnosis, first antipsychotic prescription and at index date), geographical region, area-level deprivation, comorbidities (cerebrovascular disease, dyslipidaemia, myocardial infarction, hypertension, liver disease, renal disease, diabetes, alcohol misuse, substance misuse, polycystic ovary syndrome), concomitant medication prescriptions (antidepressants, lipid-regulating medications, insulin), biochemical parameters (HbA1c, random glucose), smoking status, body mass index (BMI) and body weight.

For patients in England, if ethnicity was not coded in the primary care record, we supplemented it with ethnicity data from linked HES records, where available. If a patient had more than one ethnicity category recorded, we used the most frequent (or the most recent, if frequencies were equal). Geographic region refers to the patient’s registered primary care practice and includes Northern Ireland, Scotland, Wales and nine regions across England as per the Office for National Statistics categories. For patients in England, relative deprivation was derived from linked small area-level data using the 2019 English IMD, based on either the patient’s residential postcode or, if unavailable, the primary care practice postcode. If a patient had multiple SMI diagnoses recorded, then the most recent diagnosis category as at the index date was used, retaining the first diagnosis date. Binary indicators were used to define antidepressant prescriptions (as categorised by the British National Formulary) and insulin prescriptions in the 2 years prior to the index date. BMI (kg/m^2^) was categorised as underweight (<18.5), healthy weight (≥18.5 to <25), overweight (≥25 to <30) and obese (≥30).

### Missing data

Missing weight values and covariates were handled using multiple imputation by chained equations using the ‘mice’ package in R. The imputation model included baseline, outcome and auxiliary variables and we assumed data were missing at random. We generated 25 imputed datasets and pooled results across imputed datasets according to Rubin’s rules.

### Statistical analysis

All analyses were conducted in R (V.4.4.1). The full analytical code is available at: https://github.com/Alvin-RB/antipsychotic_metformin_coprescription.

To determine the incidence and prevalence of metformin prescription (objective 1), we first calculated the number of patients newly prescribed metformin in the following time-windows: ≥1 month before the incident SGA, ≤1 month before to ≤2 years after incident SGA, ≥2 years after the incident SGA. We then estimated the cumulative incidence of metformin co-prescription, using the Kaplan-Meier method to model the time to first metformin prescription among patients not previously prescribed metformin >1 month prior to the index date. To ensure that patients who were prescribed metformin on, or within 1 month prior to, the index date were included, we set their follow-up time at 0.5 days. For all other patients, follow-up time was censored at the earliest date of: first metformin prescription, death, end of primary care registration, last data collection from the primary care practice or completion of 2 years follow-up from index date. To examine prescribing prevalence over time, we calculated annual prevalences (standardised per 1000 patients). The numerator was the total number of unique patients with at least one metformin prescription each year from 2005 to 2017, and the denominator was the total number of eligible patients alive and remaining in follow-up during the given year.

To compare the demographic and baseline characteristics of patients who were prescribed metformin and those who were not (objective 2), we stratified descriptive statistics of the two groups by metformin exposure status. The first group comprised patients who had never been prescribed metformin, referred to as the SGA-only group and the second group comprised patients first prescribed metformin ≤1 month prior to or ≤2 years after the initiation of the SGA, referred to as the SGA+metformin group. Similarly, to investigate the potential rationale for prescribing, we compared the characteristics of patients in the SGA+metformin group with and without a recorded potential indication for metformin (ie, diabetes and/or PCOS).

To describe changes in weight over time among patients prescribed and not prescribed metformin (objective 3), we plotted descriptive line graphs of the mean weight at baseline and at 6-month, 1-year and 2-year follow-up, as well as the percentage change from baseline at each follow-up time-point (all with 95% CIs). Among patients in the SGA+metformin group, we included only those who were prescribed metformin ≤1 month prior to or ≤3 months after the index date (ie, those first prescribed metformin in close proximity to SGA initiation). This time range was selected because the first outcome time-point was 6 months, and we hypothesised that metformin initiated >3 months prior to the outcome would not allow sufficient time for the medication to exert an effect. In these primary analyses, we handled missing values using multiple imputation (see ‘Missing data’ section), but we also explored the impact of missing data through an observed-values analysis.

To estimate the potential effect of metformin on weight change, we constructed linear regression models to estimate the mean difference in weight (kg) at 6 months, 1 year and 2 years postindex date. The models included an indicator for metformin status as the main exposure and weight (kg) as the outcome. In an initial model (model 1), we adjusted for the baseline weight. In model 2, we adjusted for all the confounders identified using a directed acyclic graph (online supplemental figure 2), created to determine confounders of the relationship between metformin prescription and weight change. The variables included in the models were baseline weight (kg), antipsychotic medication, sex, age at index date, ethnicity, social deprivation, prior diagnosis of diabetes and prior diagnosis of PCOS. Models were stratified by sex, as PCOS—a key confounder—only affects females. The models were parameterised with linear terms for all variables and additional quadratic terms for continuous variables. As mentioned above, among patients in the SGA+metformin group, we included only those prescribed metformin ≤1 month prior to or ≤3 months after the index date.

## Results

### Objective 1: prevalence and incidence of metformin co-prescription

**Figure 1:**
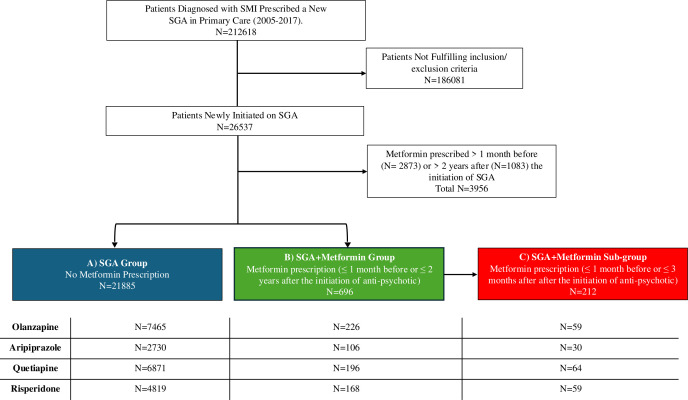
Study flow diagram. SGA, second-generation antipsychotic; SMI, severe mental illness.

Among 26 537 patients diagnosed with SMI initiating aripiprazole, olanzapine, quetiapine or risperidone, 4652 were ever prescribed metformin and 21 885 were not. A total of 696 patients were first prescribed metformin ≤1 month before to ≤2 years after first the first prescription of the SGA, 2873 were prescribed metformin ≥1 month before the SGA and 1083 were first were prescribed metformin ≥2 years after the incident SGA (figure 1, online supplemental figure 1). Among the patients not prescribed metformin within 1 month prior to the date of first prescription of the SGA (n=23 664), the cumulative incidence of first metformin prescription at 1 year was 1.9% (95% CI 1.8 to 2.1) and at 2 years was 3.3% (95% CI 3.0 to 3.5) (online supplemental figure 3). At the time-period level, period prevalence increased from 13.1 per 1000 patients (95% CI 6.5 to 19.8) in 2005 to 58.4 (95% CI 52.2 to 64.5) in 2017 (online supplemental figure 4).

### Objective 2: characteristics of patients co-prescribed and not co-prescribed metformin

Characteristics of the SGA-only and the SGA+metformin group are shown in table 1. The distribution of the types of SGAs prescribed was similar; olanzapine was the most common. The SGA-only group had a higher proportion of white individuals (84.24%) compared with the SGA+metformin group (75.23%), while the SGA+metformin group had higher percentages of Asian (11.99% vs 6.42%) and Black (10.12% vs 6.23%) individuals. The SGA+metformin group had higher levels of social deprivation, with 34.41% residing in the most deprived IMD quintile compared with 27.37% in the SGA-only group. With respect to SMI diagnosis, schizophrenia (25.29% vs 18.87%) was more common in the SGA+metformin group, while other non-organic psychoses (38.25% vs 33.91%) and bipolar disorder (42.88% vs 40.80%) were more common in the SGA-only group. The groups had similar ages at first SMI diagnosis, first antipsychotic prescription and at index date and there were limited geographical differences.

**Table 1:** Characteristics of patients prescribed an SGA (only) compared with patients prescribed an SGA and metformin (≤1 month before to ≤2 years after SGA prescription)

Characteristic	SGA only N=21 885	SGA+metformin N=696
Gender, n (%)		
Female	12 130 (55.43%)	372 (53.45%)
Male	9755 (44.57%)	324 (46.55%)
Age, mean (SD)		
At index date	53.73 (18.00)	53.57 (14.28)
At first SGA prescription	50.95 (17.87)	50.51 (14.34)
At first SMI diagnosis	45.64 (19.01)	44.90 (15.75)
Ethnicity, n (%)		
Asian	1256 (6.42%)	77 (11.99%)
Black	1218 (6.23%)	65 (10.12%)
Mixed/Other	608 (3.11%)	17 (2.64%)
White	16 476 (84.24%)	483 (75.23%)
*Unknown*	2327	*54*
SGA initiated at index date, n (%)		
Aripiprazole	2730 (12.47%)	106 (15.23%)
Olanzapine	7465 (34.11%)	226 (32.47%)
Risperidone	4819 (22.02%)	168 (24.14%)
Quetiapine	6871 (31.40%)	196 (28.16%)
SMI diagnosis, n (%)		
Bipolar disorder	9384 (42.88%)	284 (40.80%)
Other non-organic psychoses	8372 (38.25%)	236 (33.91%)
Schizophrenia	4129 (18.87%)	176 (25.29%)
Geographical region, n (%)		
East Midlands	437 (2.00%)	13 (1.87%)
East of England	911 (4.16%)	26 (3.74%)
London	4217 (19.27%)	162 (23.28%)
North East	676 (3.09%)	18 (2.59%)
North West	3597 (16.44%)	122 (17.53%)
Northern Ireland	517 (2.36%)	10 (1.44%)
Scotland	1608 (7.35%)	40 (5.75%)
South East	3416 (15.61%)	94 (13.51%)
South West	1903 (8.70%)	59 (8.48%)
Wales	1220 (5.57%)	44 (6.32%)
West Midlands	2780 (12.70%)	88 (12.64%)
Yorkshire & The Humber	603 (2.76%)	20 (5.63%)
2019 English IMD quintile, n (%)		
1 (Least deprived)	2568 (14.23%)	57 (9.66%)
5 (Most deprived)	4939 (27.37%)	203 (34.41%)
*Unknown*	*3842*	*106*
Baseline weight (kg), mean (SD)	76.83 (19.22)	90.39 (23.47)
Baseline BMI*, n (%)		
Underweight	615 (3.69%)	7 (1.22%)
Healthy	5849 (35.10%)	79 (13.72%)
Overweight	5539 (33.24%)	146 (25.35%)
Obese	4662 (27.97%)	344 (59.72%)
*Unknown*	*5220*	*120*
Comorbidities, n (%)		
Cerebrovascular disease	1439 (6.58%)	43 (6.18%)
Myocardial infarction	710 (3.24%)	27 (3.88%)
Liver disease	471 (2.15%)	17 (2.44%)
Renal disease	2235 (10.21%)	48 (6.90%)
Hypertension	5951 (27.19%)	273 (39.22%)
Dyslipidaemia	3983 (18.20%)	169 (24.28%)
Diabetes	2072 (9.47%)	339 (48.71%)
Polycystic ovarian syndrome‡	286 (2.36%)	21 (3.02%)
Other medications prescribed in **the** prior 2 years, n (%)		
Antidepressants	12 972 (59.27%)	427 (61.35%)
Lipid-regulating medications	5360 (24.49%)	293 (42.10%)
Insulin	287 (1.31%)	25 (3.59%)
Prior exposure to substances, n (%)		
Alcohol misuse	2436 (11.13%)	65 (9.34%)
Substance misuse	1823 (8.33%)	51 (7.33%)
Ex-smoker	3700 (16.99%)	104 (15.03%)
Current smoker	9383 (43.07%)	314 (45.38%)
Biochemical parameters,† mean (SD)		
HbA1c	39.06 (10.35)	54.65 (20.02)
*Unknown*	*14 968*	*264*
Glucose	5.30 (1.58)	7.61 (3.70)
*Unknown*	*4666*	*112*

Percentages are of patients with non-missing data.

* BMI (kg/m2) categories: underweight (<18.5), healthy weight (≥18.5 to <25), overweight (≥25 to <30), obese (≥30).

† Biochemical parameters are defined according to test result values recorded on or within the 2 years prior to the index date, using the value closest to the index date. For values requiring a blood test for measurement, values recorded up to 7 days after the index date were also considered on the assumption that results might relate to the date on which test results were received, rather than the date on which the blood test was taken.

‡ Percentages out of total number of females.

BMI, body mass index; HbA1c, glycated hemoglobin; IMD, Index of Multiple Deprivation; SGA, second-generation antipsychotic; SMI, severe mental illness.

The SGA+metformin group had a higher mean (SD) baseline weight (90.39 (23.47) kg) compared with the SGA-only group (76.83 (19.22) kg). With regard to BMI, 27.97% of the SGA-only patients were considered obese. In the SGA+metformin group, 59.72% of patients were considered obese. The SGA+metformin group had more patients diagnosed with dyslipidaemia (24.28% vs 18.20%), hypertension (39.22% vs 27.19%) and diabetes (48.71% vs 9.47%). Across the groups, the proportions of individuals with previous myocardial infarction, cerebrovascular disease, renal disease and liver disease were broadly similar, as were smoking status, alcohol misuse and substance misuse. A higher proportion of individuals in the SGA+metformin group were prescribed lipid-lowering drugs (42.10% vs 24.49%) and insulin (3.59% vs 1.31%), although the groups had similar proportions of patients prescribed antidepressants.

When stratified by recorded potential indications for metformin, 355 (51.0%) individuals in the SGA+metformin group had a record of diabetes and/or PCOS, while 341 (49.0%) did not. Among the 341 patients without a recorded diagnosis of diabetes or PCOS, a higher proportion were overweight (65.50% vs 55.03%) compared with those with a recorded potential indication for metformin (online supplemental table 2).

### Objective 3: changes in body weight over time

Of the 696 patients in the SGA+metformin group, 212 were prescribed metformin ≤1 month prior to or ≤3 months after the index date (ie, they were first prescribed metformin in close proximity to the initiation of SGA). These 212 patients were included in the analyses for this objective and were compared with the 21 885 patients who were prescribed SGA-only.

**Figure 2:**
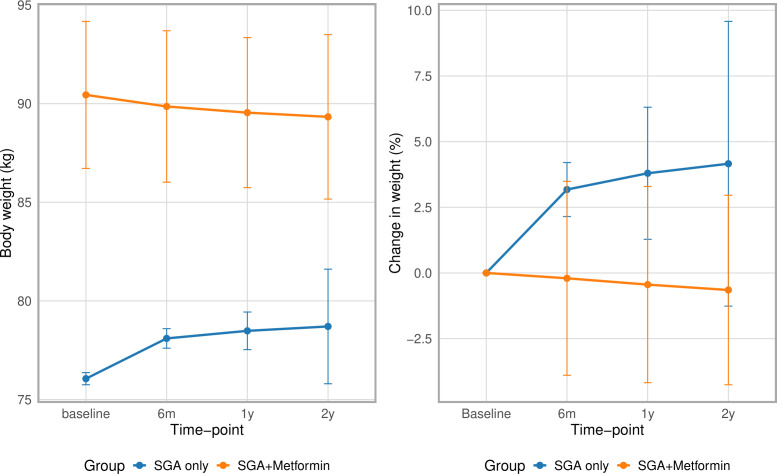
Mean absolute weight and mean percentage change in weight over time in patients prescribed SGA only (n= 21,885) vs those prescribed SGA+metformin (initiated ≤1 month before or ≤3 months after SGA prescription) (n=221). SGA, second-generation antipsychotic. Missing data were replaced using multiple imputation.

By 2 years post-SGA initiation, the mean body weight in the SGA-only group had changed by +4.16% (95% CI −1.26 to +9.58) compared with −0.65% (95% CI −4.26 to +2.96) in the SGA+metformin group (figure 2, online supplemental table 3, online supplemental figure 5). Patients in the SGA+metformin group continued to have greater absolute body weight than those in the SGA-only group throughout the follow-up period. Changes over time in the absolute values are shown in figure 2, online supplemental table 3, online supplemental figure 5.

Stratified by SGA, patients prescribed any of the four SGAs (without metformin) experienced weight gain over time. In contrast, in the SGA+metformin group, weight remained similar or decreased across each of the four SGAs (online supplemental figure 6-9).

**Figure 3:**
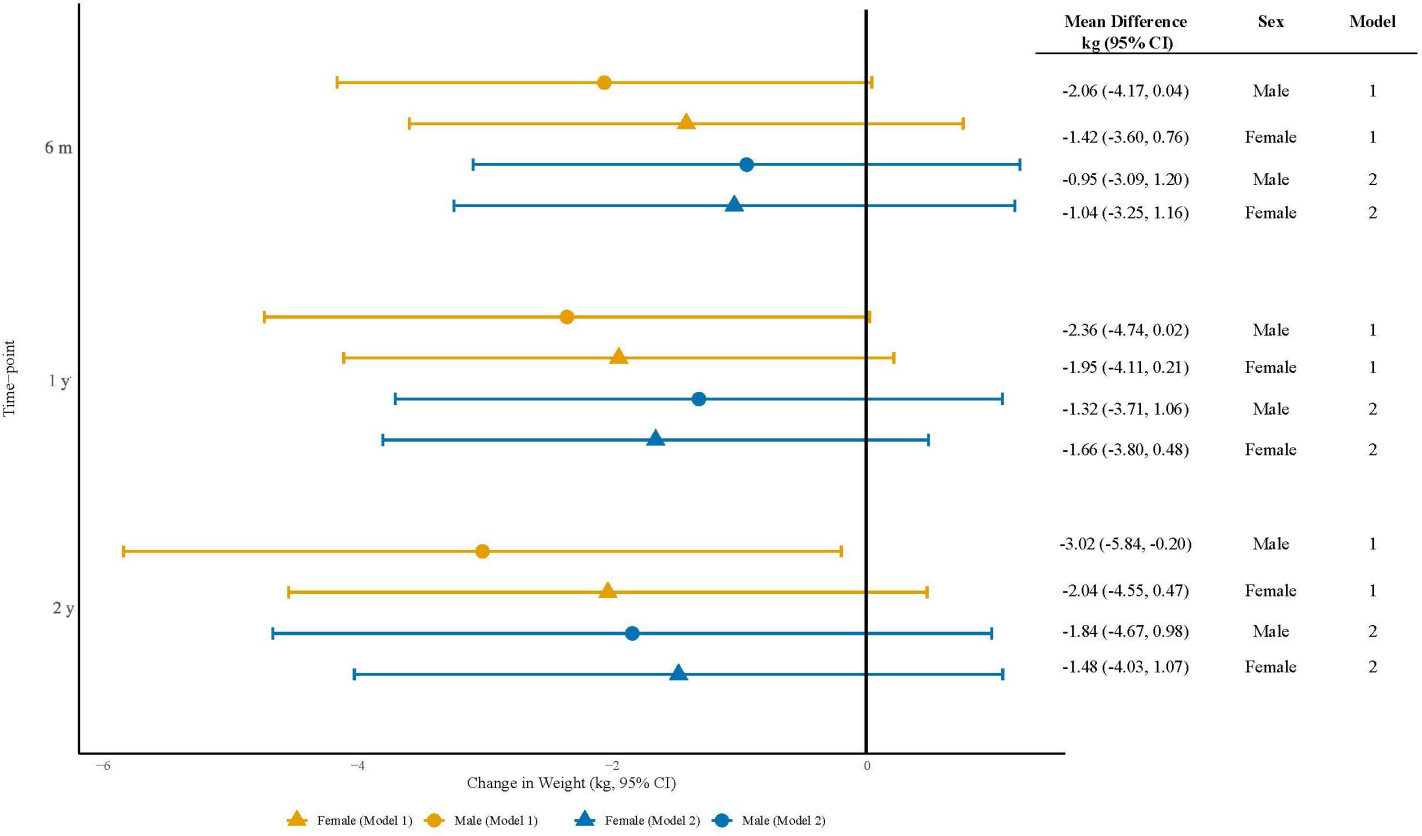
Forest plot of the estimated effect of metformin on changes in weight at 6 months, 1 year and 2 years postindex date. Model 1 is adjusted for baseline weight (linear and quadratic terms). Model 2 is adjusted for the following covariates: baseline weight (linear and quadratic terms), second-generation antipsychotic type, age at baseline (linear and quadratic terms), ethnicity, socioeconomic deprivation index, prior diabetes, polycystic ovary syndrome (for females only). Separate models were fitted for each sex. For each model, weight change was regressed on a metformin indicator variable.

When adjusting for baseline weight, the mean difference in body weight at 2 years with metformin co-prescription was −2.04 kg (95% CI −4.55 to 0.47) among females and −3.02 kg (95% CI −5.84 to −0.20) among males (figure 3). After adjusting for all specified confounders, these differences slightly attenuated; the mean difference in body weight at 2 years with co-prescription was −1.48 kg (95% CI −4.03 to 1.07) among females and −1.84 kg (95% CI −4.67 to 0.98) among males (figure 3).

## Discussion

In a large cohort of patients diagnosed with SMI, we found that few patients were co-prescribed metformin alongside the four most frequently prescribed antipsychotics in the UK (3.3% 2-year cumulative incidence). A substantial proportion of patients who were prescribed metformin had other recorded potential indications (ie, diabetes or PCOS), suggesting that metformin was not being prescribed for the prevention or management of AIWG in the majority of patients. This means that the group of patients receiving metformin specifically for AIWG is likely to be even smaller—we approximate this to be around 1% of the study cohort, raising concerns about the unmet need for metabolic risk management in this population. Perhaps promisingly, we observed that the period prevalence of metformin co-prescription rose over time (from 13.1 to 58.4 per 1000 patients); however, the absolute numbers remained very low. There did not appear to be any impact on period prevalences associated with the BAP guidance published in 2016.^21^

Through descriptive analyses, we observed a lack of weight gain in patients who were prescribed metformin ≤1 month prior to or ≤3 months after SGA initiation compared with those on SGA therapy alone. After adjustment for multiple confounders, which we identified using a direct acyclic graph, the mean difference in body weight at 2 years with co-prescription was −1.48 kg (95% CI −4.03 to 1.07) among females and −1.84 kg (95% CI −4.67 to 0.98) among males. Although our CIs are mostly consistent with a favourable effect of metformin, they also include zero, indicating uncertainty. This may be due to the relatively small number of patients co-prescribed metformin and/or the broad sample of patients diagnosed with SMI who were co-prescribed metformin in a real-world setting, whereas previous estimates largely came from short-term RCTs conducted in more restrictive settings with individuals diagnosed with schizophrenia. We therefore do not draw firm conclusions regarding the effect of metformin on weight change from our data. Instead, we recommend further studies, powered to detect smaller between-group differences than those observed in existing RCTs.^22^ However, it is important to note that patients prescribed metformin had a higher baseline weight and were more likely to have an overweight or obese BMI, potentially leading to increased non-pharmacological interventions, such as dietary and exercise advice, as well as active weight management efforts, data for which we did not have access.

Given the totality of existing evidence indicating a beneficial effect of metformin co-prescription in managing AIWG,^16^,^20^ several factors could contribute to its underutilisation. Prescribers may be deterred by potential adverse effects, such as abdominal pain, nausea, vomiting and vitamin B_12_ deficiency,^15^ particularly when these are combined with the gastrointestinal adverse effects associated with antipsychotic medications.^29^ In addition, alcohol enhances the hypoglycaemic effect of metformin and increases the risk of lactic acidosis, making metformin contraindicated for individuals with comorbid harmful alcohol use. It is recommended to monitor renal function annually and vitamin B_12_ levels in those at a higher risk of deficiency.^15^ Patients who are less likely to engage in monitoring may also be less likely to be prescribed metformin. Furthermore, AIWG is not explicitly listed as an indication in the British National Formulary, creating another barrier to its prescription for this purpose.^15^ Additionally, the NICE guidelines for psychosis and schizophrenia have not yet been formally updated. The current NICE surveillance review states that clinicians should ‘consider’ metformin as an adjunctive therapy.^23^ We were unable to ascertain how many patients had metformin considered but were subsequently not prescribed it. In addition, subsequent guidelines should clarify whether metformin should be used as a prophylactic or management strategy for AIWG. Finally, shared care of patients diagnosed with SMI between primary care and psychiatric services can lead to assumptions that the other party is managing the cardiometabolic risks of antipsychotic medications, resulting in missed interventions. A collaborative approach between both services is important to facilitate proactive monitoring and appropriate management of metabolic risks. There is also ongoing interest in other potential candidates managingAIWG. In particular, GLP-1 receptor agonists (liraglutide and exenatide) have been highlighted by NICE.^23^ Further research is needed to evaluate the real-world prescribing patterns and efficacy of GLP-1 receptor agonists in patients diagnosed with SMI.

### Strengths and limitations

This study has several strengths. To our knowledge, this is the first study to analyse metformin co-prescription in a large sample of individuals diagnosed with SMI initiating SGA therapy in UK primary care, using a data source that is representative of the UK population.^26 27^ Data generated from our study can be used to inform subsequent guideline implementation, with the potential to address cardiometabolic risks in an underserved population. Furthermore, the 12-year study period provided an opportunity to identify longitudinal prescribing trends, rather than limiting the findings to a single temporal snapshot.

Nonetheless, limitations must be acknowledged. First, the dataset included prescriptions issued only in primary care, thereby not including metformin prescribed in hospital-based outpatient clinics. Although outpatient clinics often initiate prescriptions, primary care is responsible for ongoing repeat prescriptions and so these would ultimately be represented in our data. Second, we did not have information on individual patient adherence; however, if patients are receiving repeat prescriptions, adherence could be inferred. Third, the study period concluded in 2019, and therefore did not encompass the COVID-19 pandemic, which may have impacted prescribing patterns. Fourth, although approximately 700 patients commenced metformin after SGA initiation, restricting the analysis to those who started metformin closer to SGA initiation reduced the sample size to 212. This limited statistical power and our ability to draw firm conclusions about metformin’s effect on weight change; however, this sample size still compares favourably to the largest trial of metformin in this population (n=116). Finally, our study focused on UK prescribing practices and may not be generalisable to other countries. Further studies are needed to explore the role of metformin co-prescription in other healthcare systems.

## Conclusion

Overall, these findings highlight the low utilisation of metformin, despite its apparent efficacy for AIWG, as evidenced by the existing literature and clinical guidelines. Additional well-powered studies are needed to reliably estimate the real-world effect of metformin co-prescription and to identify the patient groups that might derive the most benefit. There is also a need for clear guidance on implementing existing guidelines to ensure evidence-based care and to define the roles and responsibilities of clinicians in managing patients who experience the cardiometabolic sequelae of SGAs.

## Clinical implications

Our findings suggest the need for a proactive approach to cardiometabolic risk management through improved collaboration between primary care and psychiatric services. Enhancing prescriber awareness of metformin’s potential benefits, while addressing barriers such as off-label prescribing challenges and patient adherence, is essential. Future research should focus on the long-term effectiveness of metformin for AIWG and its integration into routine clinical practice.

## Supplementary material

10.1136/bmjment-2024-301505 Supplementary file 1

## Data Availability

Data may be obtained from a third party and are not publicly available.
